# Independent and temporally separated dynamics for RORγt and Foxp3 during Th17 differentiation

**DOI:** 10.3389/fimmu.2025.1462045

**Published:** 2025-04-28

**Authors:** Stav Miller, Inbal Eizenberg-Magar, Shlomit Reich-Zeliger, Jacob Rimer, Irina Zaretsky, Dan Reshef, Ekaterina Kopitman, Nir Friedman, Yaron E Antebi

**Affiliations:** ^1^ Department of Immunology and Regenerative Biology, Weizmann Institute of Science, Rehovot, Israel; ^2^ Department of Life Sciences Core Facilities, Weizmann Institute of Science, Rehovot, Israel; ^3^ Department of Molecular Genetics, Weizmann Institute of Science, Rehovot, Israel; ^4^ Department of Systems Immunology, Weizmann Institute of Science, Rehovot, Israel

**Keywords:** T-cell differentiation, time-lapse microscopy, transcription factor dynamics, micro-well array, double positive T-cells, systems immunology, clonal analysis

## Abstract

T helper 17 and Regulatory T cells (Th17 and Treg, respectively) are two well-described lymphocyte subsets with opposing actions. The divergent fates of Th17 and Treg cells are accounted for, at least in part, by molecular antagonism that occurs between their respective specific transcription factors, RORγt and Foxp3. An imbalance between Th17 and Treg cells may lead to tissue inflammation and is associated with certain types of autoimmunity. In order to understand the heterogeneity and dynamics of the differentiation process, we studied Th17/Treg cell differentiation of naïve cells *in vitro*, using RORγt^GFP^Foxp3^RFP^ dual-reporter mouse. Flow cytometry revealed the consistent emergence of a population of double positive RORγt^+^Foxp3^+^ (DP) cells during the early stages of Th17 cell differentiation. These DP cells are closely related to RORγt^+^ single positive (SPR) cells in terms of global gene expression. Nevertheless, for some genes, DP cells share an expression pattern with Foxp3^+^ single positive (SPF) Treg cells, most importantly by reducing IL17 levels. Using time-lapse microscopy, we could delineate the expression dynamics of RORγt and Foxp3 at a clonal level. While the RORγt expression level elevates early during differentiation, Foxp3 rises later and is more stable upon environmental changes. These distinct expression profiles are independent of each other. During differentiation and proliferation, individual cells transit between SPR, DP, and SPF states. Nevertheless, the differentiation of sister cells within a clone progeny was highly correlated. We further demonstrated that sorted SPR and DP populations were not significantly affected by changes in their environment, suggesting that the correlated fate decision emerged at early time points before the first division. Overall, this study provides the first quantitative analysis of differentiation dynamics during the generation of DP RORγt^+^Foxp3^+^ cells. Characterizing these dynamics and the differentiation trajectory could provide a profound understanding and be used to better define the distinct fates of T cells, critical mediators of the immune response.

## Introduction

CD4^+^ T cells play a central role in mediating adaptive immunity. Upon interaction of their TCR with a cognate antigen and stimulation by the cytokine milieu, naïve CD4^+^ T cells can differentiate into specialized effector subsets that help other immune cells in eradicating pathogens (1, 2). Under certain conditions, CD4^+^ T cells can instead be induced to a regulatory fate (iTreg cells), which maintains self-tolerance and modulates immune responses to infections. However, recently the paradigm of a discrete set of terminally differentiated fates has been altered, and a continuum of fates that shows a high level of plasticity was revealed (3). We and others have reported stable co-expression of multiple lineage-specific genes accompanied by a mixed cytokine secretion phenotype in committed CD4^+^ T cell populations in response to complex environmental signals (4–6). Furthermore, diverse transitions between lineages were shown to occur in both humans and mice (7–10).

An important form of plasticity is between the effector and regulatory states of T cells, enabling adaptation to changes in the cells’ environment. Transitions in both directions were described before (7, 11–13), and double positive cells co-expressing Foxp3 with each of the known effector transcription regulators RORγt, Tbet, and GATA3 were found *in vivo* and shown to possess an immunosuppressive function (14–17). In particular, the interplay between effector Th17 and Treg cells has important implications for immune responses. Upregulation of Th17 immunity and functional defects in Tregs were demonstrated in various autoimmune disorders (18–21). Treatments with a neutralizing anti-IL-17 antibody or adoptive transfer of Treg cells were able to limit disease severity in mice by restricting the expansion of effector Th17 cells (21–23). Interestingly, both Th17 and Treg cells require TGFβ for their differentiation from naïve CD4^+^ T cells and can be found in adjacency *in vivo*, for example, in the intestine (24).

**Figure 1:**
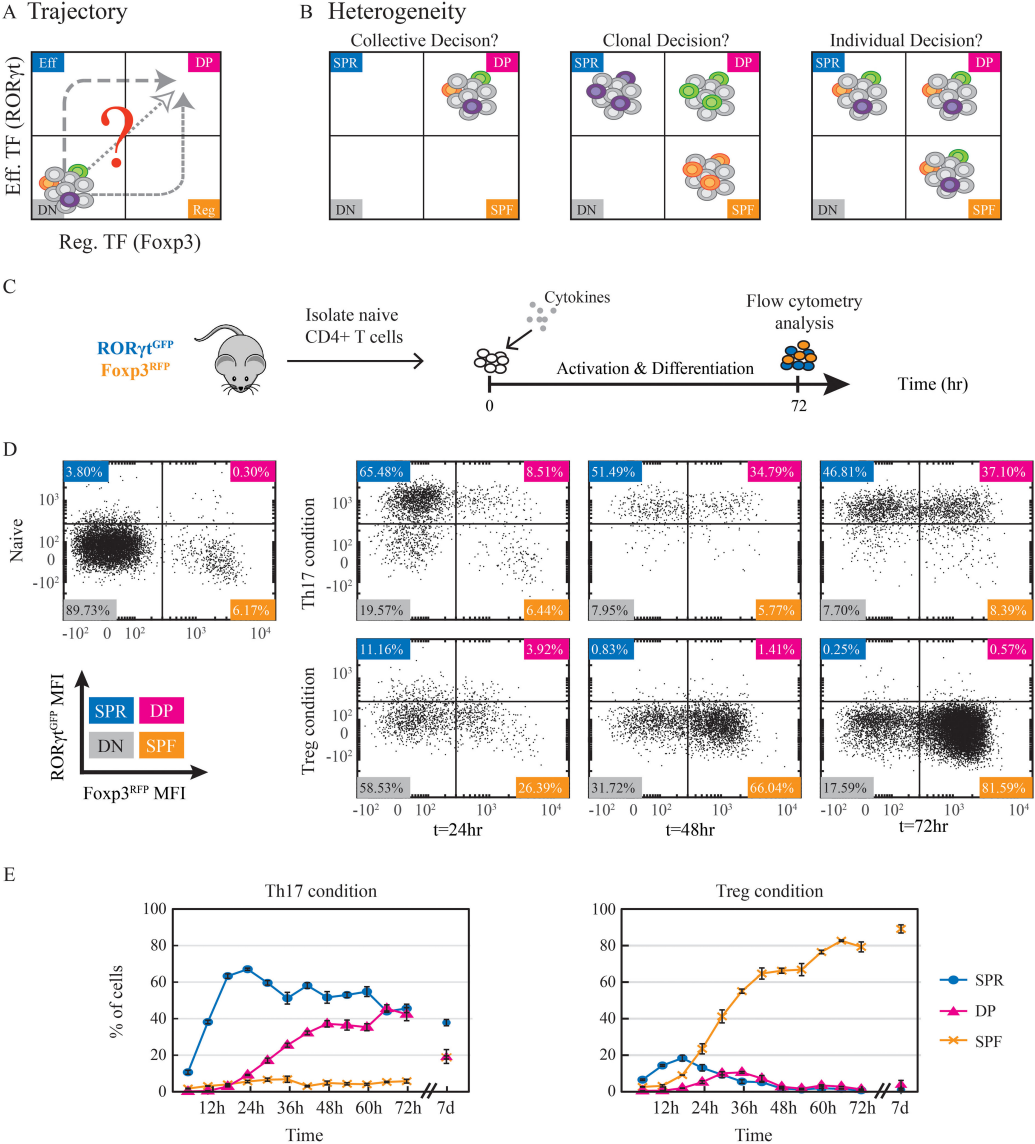
A large stable DP population emerges during Th17 cell differentiation. **(A)** Potential trajectories of naive CD4^+^ T cells towards expressing both effector and regulatory transcription factors and becoming double positive RORγt^+^Foxp3^+^ cells. **(B)** The fate decision within cells can arise at different levels. The cellular state might be the result of a collective homogeneous decision where all cells become double positive. Alternatively, some cells could attain a double positive fate, while others become single positive. In this case, the decision could occur at the clonal level, such that all the descendants of the same clone have the same fate, or each cell could make an independent individual decision. Cell color represents a clone. **(C)** Experimental design. Naïve CD4^+^ T cells were isolated from the spleen of dual reporter RORγt^GFP^Foxp3^RFP^ mice and cultured in the presence of activation beads and fate-inducing cytokines for 72hr before being analyzed using flow cytometry. **(D)** Scatter plots showing measured expression patterns of RORγt^GFP^ and Foxp3^RFP^ of naive cells on t=0 and of cells under Th17 (upper panel) and Treg (lower panels) conditions on t=24,48,72hr. Population color code: blue, SPR (single positive, Rorγt); pink, DP (double positive); red, SPF (single positive, Foxp3); gray, DN (double negative). **(E)** Percentages of detected populations of cells cultured under Th17 (left) and Treg (right) conditions. Measurements were taken every 6hr throughout 72hr and after 7 days using flow cytometry. The presented data is from one representative experiment out of three, each with three technical repeats.

While double positive RORγt^+^Foxp3^+^ cells were shown to have a significant role in regulating inflammation (14, 17, 25–27), the differentiation process of these cells has not yet been studied systematically, and its dynamics is still not known. Differentiation of double positive fates could proceed through distinct trajectories (**Figure 1A**). Double positive cells could emerge from effector RORγt^+^ cells or regulatory Foxp3^+^ cells and could differentiate directly from naive cells. Furthermore, heterogeneity in the differentiation process is a key component in determining the balance between effector and regulatory functions. The level of heterogeneity is set by whether the resulting differentiation decision is determined collectively at the population level, individually at the single cell level, or at the clonal level (**Figure 1B**). Finally, it is important to determine the stability and plasticity of the resulting fates.

## Results

### Analysis of RORγt and Foxp3 dynamics during *in vitro* Th17 cell differentiation reveals a large and stable DP population

Studying these questions requires quantitative monitoring of the dynamics of the two master transcription factors through the differentiation process at the single-cell level. We generated a tissue culture model giving rise to RORγt^+^Foxp3^+^ DP cells. Specifically, we crossed transgenic Rorc(γt)-Gfp^TG^ reporter mice with Foxp3-IRES-mRFP knock-in mice and used the resulting RORγt^GFP^Foxp3^RFP^ dual reporter F1 hybrids. To test whether we could identify double positive populations using this model, we isolated lymphocytes from Peyer’s Patches of a healthy mouse and verified the existence of a CD4^+^ DP RORγt^+^Foxp3^+^ population (14) (>3%; **Supplementary Figure S1A**).

Next, we aimed to induce double-positive cells *in vitro*. Naïve CD4^+^ T cells were extracted from the spleens of the dual reporter mice and activated using anti-CD3 anti-CD28 activation beads. The cells were incubated for three days under conditions favoring either Th17 cell differentiation (TGFβ with IL6) or iTreg (TGFβ with IL2) (**Figure 1C**). Importantly, in order to enable the emergence of mixed fates, we did not use fully polarizing conditions that include the addition of neutralizing antibodies against cytokines directing alternative fates (5). After three days, cells were measured to determine the expression of signature markers. As expected, under the Th17 condition, we observed upregulation of the master regulator RORγt and IL17A mRNA (**Supplementary Figure S1B**) as well as secretion of the signature cytokine IL17A (**Supplementary Figure S1C**). In cells cultured under iTreg-inducing conditions, on the other hand, mRNA levels of the main transcription factor Foxp3 increased (**Supplementary Figure S1B**). We then used flow cytometry to measure the expression of these two transcription factors at the single-cell protein level. Treg-inducing conditions resulted in the gradual emergence of a Foxp3^+^ population during the first three days (**Figure 1D**, lower panel). Under Th17-inducing conditions, however, we observed both a SP RORγt^+^ population and a DP RORγt^+^Foxp3^+^ population (**Figure 1D**, upper panel). We further validated the RORγt^GFP^ and Foxp3^RFP^ reporters by sorting positive and negative populations and staining them with antibodies against RORγt and Foxp3 (**Supplementary Figure S1D**). Thus, inducing CD4^+^ cells with TGFβ and IL6 *in vitro* results in a significant population of DP RORγt^+^Foxp3^+^ cells. This *in vitro* system for the differentiation of double-positive cells allows us to quantitatively study the dynamics of the differentiation process. Using flow cytometry, we measured every six hours the percentages of cells in three distinct populations: single positive RORγt cells (SPR), single positive Foxp3 cells (SPF), and double positive cells expressing both transcription factors (DP). We found that under both Th17 and Treg conditions, cells began to elevate the level of RORγt within 12hr after they encountered TCR activation and TGFβ (**Supplementary Figure S1E**). The expression of Foxp3, however, was relatively delayed and occurred at 24-36hr. Under Treg condition, the expression of RORγt was transient and disappeared within the first two days of activation (<4% of the cells). The SPF population reached a peak of 80% after 66hr (**Figure 1E**) and was stably maintained throughout an extended culture of 7 days. In contrast, under Th17-inducing condition, the fraction of cells expressing RORγt increased monotonically, reaching its maximum level of 70-90% at approx. 30hr (**Supplementary Figure S1E**). The percentage of RORγt^+^ cells remained at this level until the end of the experiment on day 7. Here, the increase in Foxp3 resulted in a population of DP cells that was stable throughout the experiment and occurred at a significant percentage (10-45%; **Figure 1E**). Importantly, under the Th17 condition, all Foxp3^+^ cells were in the DP population, and we did not observe any SPF population. To determine the role of TGFβ in the emergence and long-term stability of the DP population, we activated T-cells and cultured them under alternative Th17-inducing conditions without TGFβ. Under these conditions, no DP population was detected after 72 hours (**Supplementary Figure S1F**). In contrast, when TGFβ was included, the DP population remained stable even after TGFβ was washed out and the cells were cultured for an additional four days (**Supplementary Figure S1G**). These findings indicate that while TGFβ is required for the initial emergence of the DP population, its maintenance is independent of continuous external TGFβ signaling. Overall, we have established a tissue culture model in which we can observe the emergence of a population of DP cells. The DP cells show transient dynamics when cultured in the presence of cytokines which favor the differentiation of Treg, and long-term stable dynamics in Th17-favoring conditions.

### DP cells show mixed transcriptional expression patterns with many genes parallel to SPR or SPF expression patterns, while others are uniquely regulated

**Figure 2:**
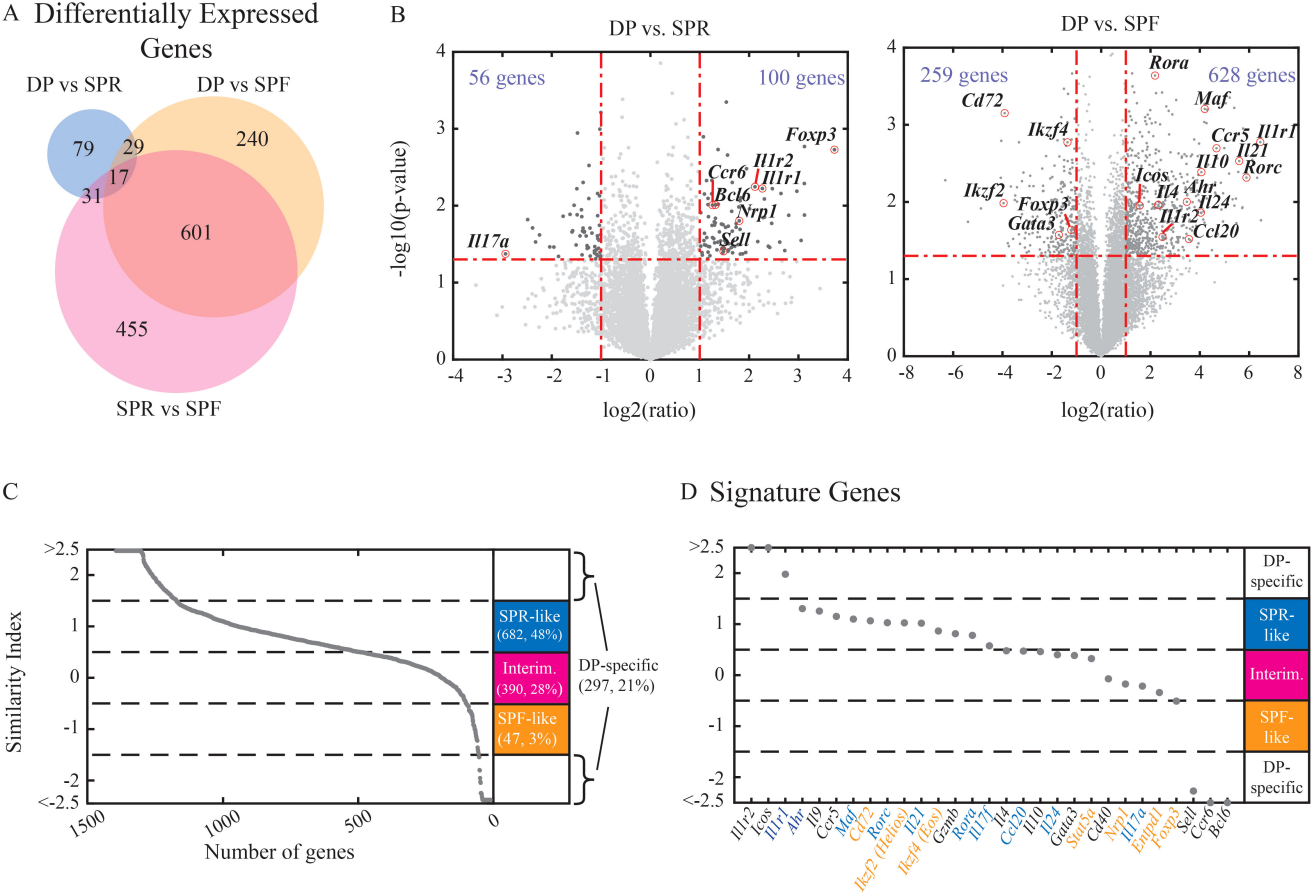
DP cells have a mixed RNA expression profile with reduced immune effector function. Naive cells were activated in the presence of Th17 or Treg-inducing conditions for 3 days and were sorted into SPR (single positive Rorγt^+^, Th17 condition), DP (double positive Rorγt^+^Foxp3^+^, Th17 condition), and SPF (single positive Foxp3^+^, Treg condition) populations. Bulk RNA was extracted for each sample and used for the construction of the RNA-seq library. **(A)** Venn diagram showing the number of differentially expressed genes between each pair of populations (fold change above 2 and Pv<0.5, a total of 1416 genes). **(B)** Volcano plots emphasize changes in specific genes while comparing DP vs. SPR (left) or DP to SPF (right). **(C-D)** Similarity index between DP to the other populations was calculated. Genes with indices between -1.5 to -0.5 were labeled as “SPF-like” (red), -0.5 to 0.5 as “intermediate” (pink), and 0.5 to 1.5 as “SPR-like” (blue). Genes below -1.5 and above 1.5 are considered to show distinct expression patterns in DP cells. **(C)** All differentially expressed genes were sorted by their similarity indices. **(D)** Immune related genes were sorted by their similarity indices (Th17- and Treg-related genes names in blue and red, respectively). An average of three independent biological repeats is presented for each sample.

Having identified distinct patterns of RORγt and Foxp3 expression, we further aimed to characterize the full transcriptional differences between the populations using RNA-seq. We analyzed the transcriptional profile of the stable DP population that emerged under Th17 condition and compared it with the SPR and SPF populations generated under Th17 and Treg conditions, respectively. Analysis of differentially expressed genes revealed significant differences between all three populations (**Figure 2A**). In particular, the DP population differed in the expression of key genes from both SPR and SPF (**Figure 2B**). Quantitatively, the comparison between SPR and SPF resulted in the largest number of differentially expressed genes, suggesting that the DP population has an intermediate transcriptional pattern. Comparing SPR or SPF to the DP population, SPR showed fewer differentially expressed genes. This finding is consistent with the fact that DP and SPR have emerged from the same Th17 culture conditions. Quantifying the full correlation between gene expression across the three populations showed a similar structure, with the DP population having an intermediate expression pattern, showing higher similarity to the SPR population than to the SPF population (**Supplementary Figure S2A**).

We then focused on the differentially expressed genes between the DP population and either the SPR or SPF populations. We quantified the expression level of each gene compared to its expression in either of the single positive populations. To do that, we calculated a similarity index where an index of 1 reflects expression levels similar to the SPR population, and an index of -1 reflects expression levels similar to the SPF population (see Materials and Methods). The DP population showed mixed expression patterns across all differentially expressed genes, where 48% of the genes had similar levels to the SPR population (**Figure 2C**). However, while both DP and SPR populations were exposed to the same culture conditions, many genes showed a distinct expression pattern in the DP population: approx. 30% of the genes showed expression at an intermediate level between SPR and SPF, while approx. 20% of the genes were up- or down-regulated in relation to both SPR and SPF, thus resulting in a distinct expression pattern that is unique to the DP population.

The DP population expressed high levels of Th17-associated genes like Rorc, Ahr, and Il21 (**Figures 2B, D**). Simultaneously, the expression of the Th17-associated effector cytokines Il17a and Il24 and the chemokine Ccl20 were downregulated in the DP population compared with SPR (**Figures 2B, D**). We further confirmed a corresponding reduction at the protein level of IL17A using flow cytometry (**Supplementary Figure S2B**). Since IL17 is involved in inducing and mediating proinflammatory responses (1), our data suggests that the DP population may not act as an effector Th17. Interestingly, the DP population significantly upregulated genes participating in the IL1 signaling pathway, including the activation receptor IL1R1 and the decoy receptor IL1R2 (**Figure 2D**; **Supplementary Figure S2B**). In agreement with transcriptional differences in the IL1 signaling pathways, IL-1 induces an opposite effect on the expression of IL23R, a key target of the IL1 pathways (**Supplementary Figure S2C**). The DP population also showed high expression of several Treg-associated genes, including Foxp3 and Nrp1 (**Figure 2D**). Our findings are in agreement with a recent study on *in vivo* double-positive Rorγt^+^Foxp3^+^ cells, showing a mixed transcriptional phenotype with low expression of Th17 signature cytokines (Microarray results of Il17A, Il17F, and Il21) accompanied with high expression of several Treg-associated genes (CD25, GITR (14);). Moreover, Yang et al. revealed a significant regulatory capacity using adoptive transfer of the DP cells in the context of T cell transfer colitis (14). Accordingly, using a standard *in-vitro* suppression assay, we have confirmed that DP cells grown *in vitro* under the Th17 condition can suppress the proliferation of other T cells (**Supplementary Figures S2D, E**). Overall, the DP population shows marked differences from SPR in the expression of genes with effector function and an increase in properties related to an inhibitory function.

### RORγt and Foxp3 exhibit distinct and independent temporal dynamics

**Figure 3:**
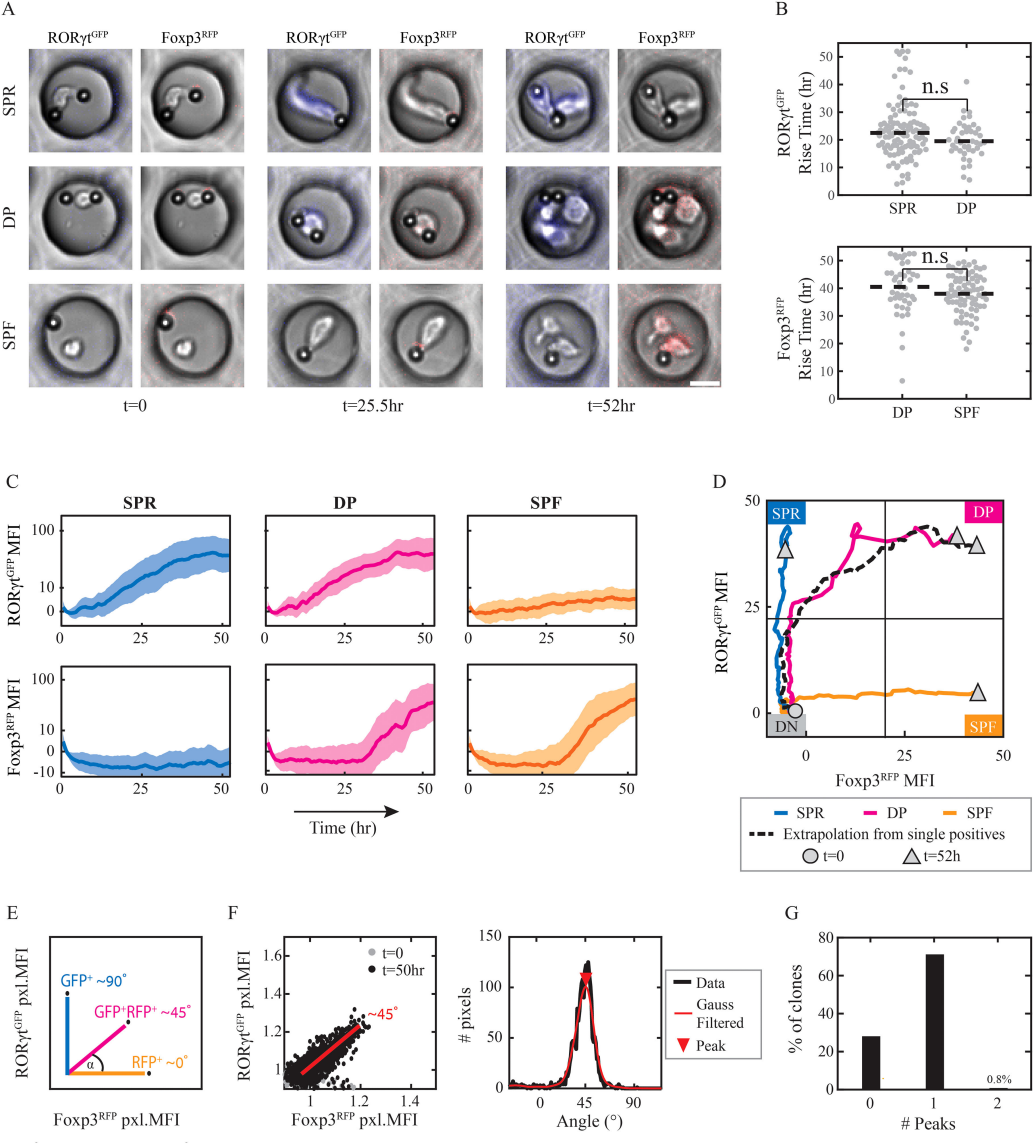
RORγt and Foxp3 expression dynamics arise at distinct timescales. Naïve CD4^+^ T cells were plated in micro-wells, supplemented with differentiating cytokines and monitored under the microscope for 52hr. Analysis was done on micro-wells with 1 cell and at least 1 activation bead at the beginning of the experiment. **(A)** Images of representative micro-wells on t=0,25.5,52hr: elevation of RORγt^GFP^ level in SPR cells under Th17 condition (top), of both RORγt^GFP^ and Foxp3^RFP^ levels in DP cells under Th17 condition (middle) or of Foxp3^RFP^ level in SPF cells under Treg condition (bottom). Scale bar, 10*μ*m. Blue, RORγt^GFP+^ pixels; Red, Foxp3^RFP+^ pixels. **(B)** Fluorescence rise time above response threshold of RORγt^GFP^ in SPR and DP clones under Th17 condition (top); and of Foxp3^RFP^ in DP clones under Th17 condition and in SPF clones under Treg condition (bottom). Dashed line, median values. A two-samples t-test was calculated for all presented pairs, Pv>0.05. Each dot represents one micro-well. **(C)** Fluorescence intensity over time of RORγt^GFP^ (upper panel) and Foxp3^RFP^ (lower panel) in SPR and DP clones under Th17 condition (left and middle, respectively) and SPF clones under Treg condition (right). A median of data is shown ± std. **(D)** Median values of traces of SPR, DP, and SPF clones drawn in the space of RORγt (y-axis) vs. Foxp3 (x-axis). Extrapolation of median values of RORγt in SPR clones vs. median values of Foxp3 in SPF clones over time is shown as a dashed line. Circular node, t=0; triangle, t=52hr. **(E)** Expected pixel angles between RORγt^GFP^ (90°) and Foxp3^RFP^ (0°) depending on fluorescence signals in 3 putative pixels. **(F)** Pixel fluorescent intensity angles in one representative DP clone: pixel angles on t=0,52hr (left); distribution of pixel angles over last 5 time frames, with Gaussian filtering and maxima local peak (red triangle) at ~45° (right). Each dot represents one pixel. **(G)** Percentages of micro-wells showing either 0,1 or 2 angles’ peaks at the last 5 time frames. The presented data is from one representative experiment out of two.

Using flow cytometry, we established the existence of the DP population. However, this data cannot determine the differentiation kinetics of the double-positive population. In particular, the initial population contained ~6% of Foxp3^+^ cells (**Figures 1D, E**), raising the possibility that some DP cells could originate from this non-naive population. Determining the DP population’s origin and kinetics requires a continuous analysis of the differentiation process. To do that, we utilized the micro-well array (MWA) (28, 29), where naïve CD4+ T cells are seeded in PDMS micro-wells (28, 29) with activation microbeads in the presence of Th17 or Treg conditions. This allowed us to monitor the cells continuously for over 2 days using a fluorescent microscope (**Figure 3A**). We note that the micro-well analysis has several key differences compared with a flow cytometry analysis. Most significantly, in the MWA, we analyze the fluorescence within each well. This allows us to follow the specific dynamics continuously and determine the complete history throughout the differentiation process. However, this analysis is done for the entire well, which generally represents an individual clone emerging from the naive cell, but not a single cell. This can affect the measured frequency of subpopulation compared with a flow analysis due to variability in death and proliferation. Another difference is the lower culture density, as cells are initially plated at an average density of a single cell per micro-well. This is a general issue for studying single cells, however, using MWA, we still allow cells to exchange signaling molecules such as cytokines as all cells share the same media. Despite these differences, we were able to use the MWA and found that the T cells grew and divided during the experiment and that clones remained within the micro-well. Overall, using the micro-well assay, we were able to follow the differentiation dynamics at a single-colony resolution for RORγt and Foxp3 expression across 378 clones during 52hr of differentiation.

To make sure we follow a complete differentiation process, we aimed to start with only cells in the naive state. We reduced initial variability by using naive CD4^+^ T cells that were extracted using a stricter extraction kit (see Materials and Methods), resulting in 97% Rorγt-Foxp3- cells (**Supplementary Figure S3A**). Furthermore, we included in our analysis only wells that were verified to have a single cell with no fluorescence in either channel at the initiation of the time-lapse experiment. We then followed the expression of the two transcription factors at 30-minute intervals and extracted the overall total fluorescence signal in the well across time in both the GFP (RORγt) and RFP (Foxp3) channels (see examples for clones in **Supplementary Figure S3B**).

To determine the differentiation outcome of each clone, we set a response threshold according to the levels of signal in naive cells at the beginning of the experiment. Rise time above this threshold enabled classification into SPR, SPF, or DP clones at the end of the experiment (52hr; **Supplementary Figure S3C**). Under the Th17 condition, we find that most cells upregulated RORγt (79%), with 57% of all clones being SPR and 22% being DP. Under Treg condition, on the other hand, 66% of the clones were Foxp3^+^: 46% of the clones being SPF, while some Foxp3^+^ cells (20%) also show a slight increase in RORγt expression. The DP clones that arise under the Treg condition expressed RORγt at a relatively low level (**Supplementary Figures S3C, D**) and do not seem to comprise a separate population. We thus focused on the DP clones arising in the Th17 condition, which comprise a distinct population and express high levels of both RORγt and Foxp3 at levels similar to the corresponding single positive populations (**Supplementary Figure S3E**).

Using the temporal data, we analyzed the dynamics of the two transcription factors. Examination of fluorescence rise time revealed a specific timescale for each of the factors, with faster dynamics for RORγt compared to Foxp3 (21.8 ± 9.4hr vs. 39.3 ± 8.2hr. Pv=2x10^-32^, **Supplementary Figure S3F**). This observation is in agreement with our flow cytometry experiments, which showed earlier expression of RORγt (**Figure 1E**). We further tested whether the two factors affect each other’s dynamics when co-expressed in DP clones. We found no significant difference in rise time for either factor in DP clones compared to the SPR and SPF clones (**Figure 3B**). Moreover, this similarity extends beyond the rise time to the entire dynamics of transcription factors expression (**Figure 3C**). The fluorescence intensity at the end of the experiment is similar for RORγt between SPR and DP (MFI=39.12 ± 2.6 vs. 41.74 ± 4.25, respectively), and for Foxp3 between SPF and DP (MFI=43.49 ± 5.6 vs. 37.42 ± 6.95, respectively; **Supplementary Figure S3E**). We found that the trajectory of the DP clones in the Foxp3-RORγt space falls precisely on top of an extrapolated trajectory combining the Foxp3 dynamics from SPF clones with RORγt dynamics from SPR clones (**Figure 3D**). Importantly, these results repeat in an independent MWA experiment of additional 1092 clones (**Supplementary Figures S3G, H**). Overall, we identify a distinct temporal separation between an early onset of RORγt and a later onset of Foxp3, where the timescale of each factor is robust and independent of the expression of the other factor.

### Analysis of intra-clonal homogeneity suggests a correlated differentiation of sister cells

Our time-lapse analysis measures expression in a single clone (micro-well) rather than tracking individual cells. Therefore, a micro-well classified as DP can either contain RORγt^+^Foxp3^+^ DP cells or multiple SP cells, each expressing either RORγt or Foxp3. To distinguish between the two options, we characterized the cell-to-cell heterogeneity within clones during the first 52 hours. For that, we calculated the ratio of GFP to RFP fluorescence for each pixel within the micro-well. We quantified the ratio by placing each pixel on a two-dimensional plot, in which the x-axis is RFP fluorescence, and the y-axis is GFP fluorescence. Pixels with solely high GFP result in angles around 90° while the angles of pixels with solely high RFP are around 0° (**Figure 3E**). The resulting distribution of angles across all pixels can be used to characterize the heterogeneity within the clone (**Figure 3F**, **Supplementary Figure S4A**). We then determined the number of peaks in the distribution using a peak detection algorithm (see Materials and Methods for full algorithm description). Homogenous clones are expected to exhibit an unimodal distribution, while clones composed of cells with distinct states are expected to exhibit multi-modality. To test the capability of our algorithm to identify heterogeneous clones, we generated artificial data by combining pairs of micro-well data from different populations to obtain pixel distributions of hypothetical heterogeneous clones (**Supplementary Figure S4B**). We find that our algorithm can identify these as multi-peak distributions. Thus, analyzing the RFP-GFP ratio for individual pixels and detecting multiple peaks within the resulting distribution allows us to determine the clonal heterogeneity within a micro-well.

Using this approach, we went on to characterize the number and position of the peaks in the full data set. Consistent with our expectations, clones classified as SPF show distributions with peaks around 0°, clones classified as SPR show peaks around 90°, and clones classified as DP show peaks at intermediate angles (**Supplementary Figure S4C**). Using our peak detection algorithm, we identified whether each clone had a single peak or multiple peaks. We found that multiple peaks were detected for less than 1% of the micro-wells suggesting that under our culture conditions, T-cell clones show homogeneous expression of RORγt and Foxp3 across all individual cells (**Figure 3G**). Intra-clonal homogeneity was further validated by manual tracking of approx. 100 of the clones (approx. 25% of micro-wells). These results imply that cells within a single clone show synchronized behavior between sister cells.

The most straightforward way to explain the observed homogeneity of fate choice within a clone is through simple inheritance. In this case, the parental cell increases the expression of the transcription factors, and all descendent cells simply inherit the fate of the parental cell. To test whether this occurs in our system, we identified the time of the first division in each micro-well by manually tracking the parental cells. Our analysis shows that the median time of the first division is later under Th17 condition compared to Treg condition (38.8 ± 0.63hr for Th17, 36 ± 0.63hr for Treg, Pv=0.005; **Supplementary Figure S5A**). In addition, the SPR population has a later division time compared with DP and SPF populations (**Supplementary Figure S5B**). When we compared the division time to the expression time of the transcription factors, we found that the expression time of RORγt (21.8 ± 0.06hr) is earlier than the first cell division in most cases, consistent with a parental fate choice that propagates to the descendants. However, the expression of Foxp3 (39.3 ± 0.07hr) occurs mostly after the first division (**Supplementary Figure S5C**). In this case, multi-cell clones up-regulate the expression of Foxp3 autonomously and simultaneously. Thus, the observed clonal homogeneity in the expression of Foxp3 cannot be explained simply by the propagation of a pre-established parental expression and, therefore, requires a different mechanism to account for the coordination of transcription factor expression.

### The cellular environment has minimal effect on the cell state

**Figure 4:**
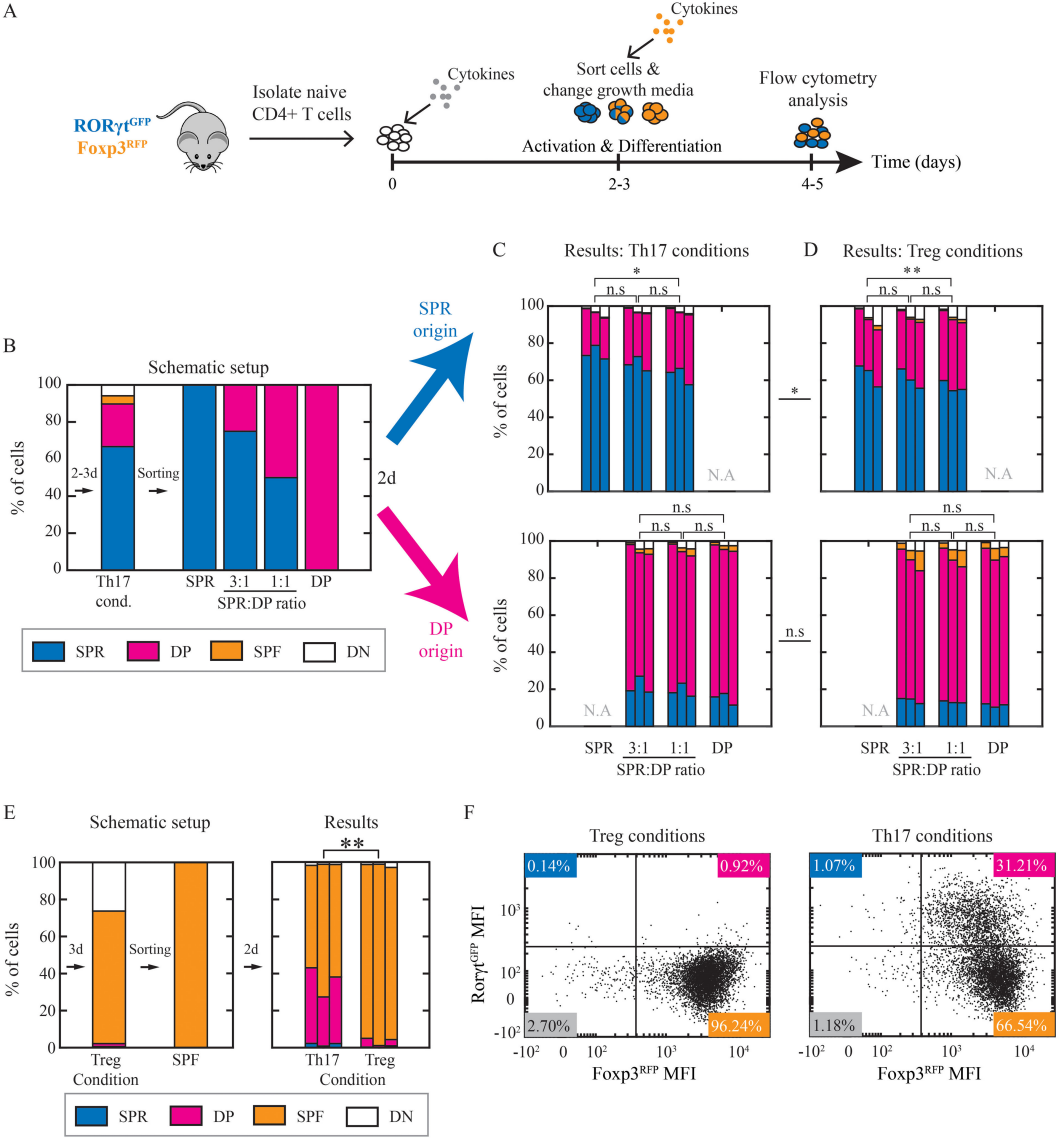
Th17 cells’ state is not affected by neighboring cells or environmental cues. **(A)** Cells were cultured under Th17 and Treg conditions for 2-3 days. Then they were sorted to SPR, DP, and SPF and re-cultured under Th17 or Treg conditions for additional 2 days (day 4 or 5 from the beginning of the experiment). **(B)** Schematic representation of experimental setup. SPR and DP cells were sorted. Then, SPR cells were stained with eFluor 450 dye to enable their detection post co-culturing. Sorted cells were re-cultured individually (100% SPR; 100% DP) or co-cultured at 3:1 (75% SPR:25% DP) and 1:1 (50% SPR:50% DP) ratios for 2 days. **(C, D)** Resulting populations (panel of 4 plots) that were SPR originated (eFluor 450^+^; upper panel) or DP originated (eFluor 450^-^; lower panel) and re-cultured under Th17 **(C)** or Treg **(D)** conditions were measured on flow cytometry. Three independent biological repeats are presented. **(E)** SPF cells were sorted after 3 days of differentiation and re-cultured under Th17 or Treg condition. Resulting populations were measured on flow cytometry following 48hr of culture. Three independent biological repeats are presented. **(F)** Scatter plots showing measured expression patterns of RORγt^GFP^ and Foxp3^RFP^ of SPF sorted cells after 2 days of re-culturing under Th17 (left) and Treg (right) conditions. Data is from one representative experiment out of three. Blue, SPR; pink, DP; orange, SPF; white, DN.

The uniform increase of Foxp3 expression across sister cells can be explained either by shared environmental factors coordinating and directing the cells or by an inherited shared internal state. We first tested whether the cellular composition within the population can direct the transition from SPR to DP cells. For that, we sorted cells cultured under Th17 condition after 48-72hr of activation to SPR and DP cells and further cultured them under fresh cytokine conditions for 2 days (**Figure 4A**). SPR cells were stained with efluor 450 to enable tracing the origin of cells after co-culturing. To vary the cellular composition, we co-cultured cells at different SPR: DP ratios under fresh Th17 conditions (**Figure 4B**). Flow cytometry analysis after two days of re-culture revealed transitions in both directions. We found DP cells that originated from SPR cells and vice versa when culturing each population alone (transition from SPR to DP 21.7 ± 3.8% and from DP to SPR 15.01 ± 3.22%; **Figure 4C**). As we vary the SPR: DP cell number ratio, we find a minimal change in the transition frequency (**Supplementary Figures S6A, B**) which could not explain the high level of homogeneity observed.

We next tested whether extracellular environmental signals can induce a transition to DP cells. To do that, we repeated the co-culture experiment and changed the culture conditions from Th17 to Treg conditions following cell sorting (**Figure 4D**). Similarly to the previous results, the transition frequencies between SPR and DP show only a minimal difference (**Supplementary Figures S6A, B**). Similar results were obtained after 4 days of re-culturing instead of 2 days (**Supplementary Figures S6C, D**). We thus concluded that both the composition of neighboring cells and the environmental cues did not significantly affect the transition in the expression of Foxp3, suggesting that this transition is mainly a predetermined cell-inherent property.

We continued to test the stability of the expression of transcription factors in the SPF population. We sorted SPF cells after 72hr of activation and re-cultured them under Treg or Th17 condition for an additional 48hr. Under Treg condition, most cells remained in the SPF population (96.2%; **Figures 4E, F**). In contrast, changing to Th17 conditions led to a reduction in the fraction of SPF cells, as approx. 30% of the cells significantly up-regulated RORγt (**Figures 4E, F**, **Supplementary Figure S6E**), resulting in DP cells that originated from SPF cells. We note that this did not result in an increase in the SPR population, as the expression of Foxp3 was stable. These results support the idea of partial cell plasticity and the capacity for reprogramming during Treg differentiation via extracellular control of expression levels of RORγt, while the Foxp3 expression is more robust to extracellular signals.

## Discussion

Double positive RORγt^+^Foxp3^+^ cells comprise an important T helper (Th)-cell state that has been thoroughly studied *in vivo*. However, many aspects of its differentiation dynamics are still unknown. Using an *in vitro* system based on dual-reporter RORγt^GFP^Foxp3^RFP^ mice, we have identified conditions that give rise to distinct double-positive populations *in vitro*. When naive CD4^+^ T-cells are cultured with TGFβ and IL6, a large double positive population emerges that is stable for at least 7 days. When cultured with TGFβ and IL2, classic Treg-inducing conditions, a small proportion of cells increased RORγt levels at low levels, with most cells becoming Foxp3^+^ Treg cells. Finally, another double positive population can be generated by trans-differentiation of the Foxp3^+^ cells in the presence of TGFβ and IL6.

Distinct populations of RORγt^+^Foxp3^+^ cells were also identified *in vivo* (14, 17, 25), showing some phenotypic differences. In some studies, DP cells were found to be in an intermediate state, being able to terminally differentiate into single-positive RORγt^+^ (SPR) or single-positive Foxp3^+^ (SPF) (25, 30). In other work, a stable *in vivo* DP population with enhanced suppressive capacity was identified (14). However, DP cells show a distinct expression of surface molecules and transcription factors from those of Th17 and Treg cells. Our *in vitro* system shows similarities with these patterns, along with a suppressive capacity, and can further enable the study of distinct differentiation trajectories that all lead to the DP phenotype.

Using our *in vitro* differentiation protocol, we quantified the dynamics of double-positive cell differentiation. We identified a separation of timescales between the rapid response of the effector transcription factor RORγt and the delayed increase of the regulatory transcription factor Foxp3. The difference in dynamics between the two transcription factors was independent and showed the same trajectory in single-positive and double-positive cells. Transcriptional analysis suggests a reduced effector function for the double positive population, in particular, lowered expression of IL17. This reduced expression is consistent with previous data showing that Foxp3 binds to RORγt and suppresses IL17 transcription (25, 31–33). Additionally, the regulatory function for DP cells grown under Th17 condition was confirmed using a suppression assay. The separation of timescales between Rorγt and Foxp3 expression suggests a dynamic process at the population level. Cells first reach an effector state and start to induce an immune response. Only then does a fraction of the cells increase Foxp3 levels, which inhibits their effector function and puts them in a suppressive state, where they play a role in shutting down the immune response. This dynamic is reminiscent of an incoherent feed-forward loop (IFFL) (34) in genetic networks, where a protein has a fast positive regulatory effect and a slow inhibitory function. It was found that IFFLs can provide a transient, adaptive response to signals and it would be interesting to further study whether similar roles can be identified for the delayed Foxp3 activity.

Finally, we studied the stability of the Th cell populations. While the Foxp3 response occurs later, we found that it is homogeneous across cells within the same clone, suggesting an early decision. Accordingly, external cytokines and cellular composition did not further affect Foxp3 expression. In contrast, RORγt exhibits a more flexible behavior, as changing the signaling environment can induce its expression even at late time points.

Our findings shed new light on the development and plasticity of double positive RORγt^+^Foxp3^+^ cells. We demonstrate that the DP state can emerge under several conditions with different dynamics. The largest population of DP cells is generated during Th17 cell differentiation and exhibits a stable phenotype. Deciphering the cell state composition is important for putative therapeutic manipulations aimed to shape the balance between Th17 and Treg cells in autoimmune diseases.

## Data Availability

RNA-seq data have been deposited to Gene Expression Omnibus (GEO) with the accession no. GSE227988. Imaging data from micro-wells experiments is available in DOI: 10.34933/4fbfe367-86b8-431e-92f2-94c5cb3465f5.
