# Supplementary material for: Independent and temporally separated dynamics for RORγt and Foxp3 during Th17 differentiation
Source: Front Immunol. 2025 Apr 28;16:1462045. doi: 10.3389/fimmu.2025.1462045 (PMC12066577; doi:10.3389/fimmu.2025.1462045)
Supplement: Supplementary file 1 [file DataSheet1.docx]

Supplementary Materials for

**Independent and temporally separated dynamics for RORγt and Foxp3 during Th17 differentiation**

Stav Miller, Inbal Eizenberg-Magar, Shlomit Reich-Zeliger, Jacob Rimer, Irina Zaretsky, Dan Reshef, [Ekaterina Kopitman](https://www.weizmann.ac.il/pages/search/people?keywords=ekaterina), Nir Friedman, Yaron Antebi

Corresponding authors: [yaron.antebi@weizmann.ac.il](mailto:yaron.antebi@weizmann.ac.il)

**The PDF file includes:**

Materials and Methods

Tables S1 to S2

Figs. S1 to S6

**Materials and Methods**

Mice

Transgenic Rorc(γt)-GfpTG reporter mice (generated in Gerard Eberl’s lab; [(35)](https://paperpile.com/c/liLVgS/3CNfd)) were a gift from Eran Elinav. Female Foxp3-IRES-mRFP knock-in mice (strain #008374) and B6SJL mice were purchased from Jackson laboratory. The resulting RORγt^GFP^xFoxp3^RFP^ males of F1 generation are heterozygous for both reporters. Mice were housed under specific pathogen-free conditions at the animal facility of the Weizmann Institute and used at 6–10 wk of age. All animal experiments were performed under protocols approved by the Animal Care and Use Committee of the Weizmann Institute.

*In vitro* differentiation

Naïve CD4^+^ T cells were isolated from spleens of mice by magnetic microbeads (CD4^+^CD62L^+^ T cell isolation kit, mouse; Miltenyi Biotec) and MACS separation columns, according to the manufacturer’s instructions. Cells were cultured in RPMI supplemented with 10% FCS at a concentration of 1X10^6^ cells/ml. For stimulation, cells were activated with anti-CD3 anti-CD28 activation beads (Dyanbeads) at a 1:1.25 beads to cells ratio. For *in vitro* differentiation, cells were supplemented with TGFβ (5ng/ml) and IL6 (20ng/ml) to induce Th17 cells or TGFβ (10ng/ml) with IL2 (5ng/ml) to induce Treg cells, and cultured for 3 or 5 days, as indicated. An alternative Th17-inducing condition without TGFβ (fig. S1F,G) included IL6 (20ng/ml), IL1β (20ng/ml) and IL23 (50ng/ml). Culturing with activation beads and IL2 (5ng/ml) was used as a control. Longer incubation of 7 days (Fig. 1E) included: Th17 or Treg cell differentiation in the presence of cytokines and activation beads for 3 days, separating beads from cells and leaving at rest for 4 more days (in case of Treg cells, adding 5ng/ml IL2). Cytokines: rmIL-2 (R&D, 402-ML-020), rhTGFβ1 (R&D, 240-B-002), mIL6 (R&D, 406-ML-005), mIL1β (Peprotech, 211-11B-2UG), hIL23 (Peprotech, 200-23-10UG).

Flow cytometry

For the detection of Il17 cytokine (fig. S2B), cells were supplemented with Brefeldin A and Monensin (Biolegend) for 2 hours in growth media prior to sample collection. For all panels, to separate between cells and activation beads, the growth plate was placed on a vortex for 30 sec and then on a magnet for 2-3 minutes. Cells were then collected into a fresh tube and washed with PBS. For kinetics assay (Fig. 1D,E, fig. S1E) cells were stained with blue fixable Live/Dead for the detection of dead cells and PB anti-CD4 for 30 min at R.T (see a detailed staining panel in supplementary table 1). For the detection of intracellular proteins (RORγt, Foxp3, Il17; fig. S2B, S1D), cells were first stained with Zombie aqua viability dye and anti-CD4 for 30 minutes at R.T. Then, cells were fixated using Foxp3 fixation/permeabilization buffer (eBioscience) according to the manufacturer’s instructions and permeabilized using Permeabilization buffer (10X) (Biolegend). Cells were then stained with anti-RORγt, anti-Foxp3, anti-IL1R1, anti-IL1R2 or anti-IL17 for 30 min at R.T. Samples were measured using the BD FACS LSRII (BD Biosciences) and the Cytek Aurora (Cytek Biosciences) flow cytometers with 5 lasers, and data were analyzed using MATLAB.

**Table S1.** Flow cytometry staining dyes and antibodies

| *Staining* | *Fluorophore* | *Company* | *Antibody clone/cat#* | *Dilution* |
| --- | --- | --- | --- | --- |
| Viability | Live\dead fixable blue | Invitrogen | - | 1:1000 |
| Viability | Zombie aqua fixable | Biolegend | - | 1:500 |
| anti-CD25 | PE | eBioscience | clone PC61.5 | 1:100 |
| anti-CD4 | PB or APC/Cy7 | Biolegend | clone RM4-5 | 1:200 |
| anti-IL1R1 | APC | Biolegend | clone JAMA-147 | 1:20 |
| anti-CD45.1 | PE-Cy7 | Biolegend | clone A20 | 1:100 |
| anti-CD45.2 | PerCP | Biolegend | clone 104 | 1:100 |
| anti-IL1R2 | BV421 | BD | clone 4E2 | 1:20 |
| anti-RORγt | PE | Invitrogen | clone B2D | 1:80 |
| anti-Foxp3 | AF647 | Biolegend | clone 150D | 1:20 |
| anti-Il17a | AF488 | Biolegend | clone TC11-18H10 | 1:80 |
| Proliferation | efluor 450 cell proliferation dye | Invitrogen | 65-0842-85 |  |
| Annexin V | APC | Biolegend | 640920 | 1ul/sample |
| 7-AAD | ex.488/em.650nm | Biolegend | 420403 | 1ul/sample |

Sorting cells to populations

Following three days of differentiation under Th17 or Treg conditions, cells were separated from activation beads. Cells were collected, stained with LIVE/DEAD fixable blue and anti-CD4 for 20-30 min at R.T and washed. Analysis of cells was done in BD FACS AriaIII 5-lasers cytometer (BD Biosciences). Gated CD4^+^ live cells were further grouped and sorted based on their reporters’ staining: RORγt^GFP+^ (SPR), RORγt^GFP+^Foxp3^RFP+^ (DP) and Foxp3^RFP+^ (SPF) populations. Secondary culture of the sorted groups was carried out for either restimulation to enable IL17 detection (fig. S2B), or for additional 2-days culturing with fresh Th17 and Treg-inducing cytokines to test the effect of environment on cells’ state (Fig. 4). For co-culturing of populations, SPR cells were stained according to manufacturer’s instructions with eFluor 450 dye (Invitrogen) post sorting and prior to culturing.

Suppression assay

Naive CD4^+^ T cells were isolated from dual reporter Rorgt^GFP^Foxp3^RFP^ mice (CD45.2^+^) and undergone an in vitro differentiation towards Th17 cells for 4 days. Cells were stained with blue fixable Live/Dead for 30 minutes and then sorted to extract the DP population. For the isolation of nTregs (Natural T Regulatory cells), splenocytes from a Foxp3^GFP^ mouse (CD45.2^+^) were extracted and stained with blue fixable Live/Dead, APC-Cy7 αCD4 and PE αCD25 for 30 minutes and then sorted for CD4^+^Foxp3^+^CD25^+^ cells. Another fraction of CD4^+^Foxp3^-^ cells was sorted as a negative control for the suppression assay. For T Responder cells (Tresp), naive cells were isolated from B6SJL mice (CD45.1^+^) using magnetic microbead negative separation (Naïve CD4^+^ isolation, StemCell Technologies). Tresp cells were stained with efluor 450 to enable further tracking of divisions. To obtain APCs (Antigen Presenting Cells), splenocytes isolated from B6SJL mice were irradiated at 3000 rad, centrifuged and re-cultured in fresh RPMI. Tresp cells were seeded at 50K cells/well inside a 96w plate and supplemented with 50K APCs and 1μg/ml anti-CD3 (Biolegend, clone 145-2C11) for activation. To test suppression, nTregs, DP or Foxp3^-^ cells were added with Tresp at a ratio of 1:1, and cultured for 4 days. For flow Cytometry analysis, cells were washed in  PBS Ca^+^Mg^+^ (requires for Annexin V detection), and stained with PE-Cy7 anti-CD45.1, PerCP anti-CD45.2, APC-Cy7 CD4 and APC anti-Annexin V for 30 minutes. Samples were stained with 7AAD right before readout in the Cytek Aurora (Cytek Biosciences) flow cytometery with 5 lasers. Data were analyzed using FlowJo. Proliferation index for Tresp cells was calculated as the total number of divisions divided by the number of cells that went into division.

ELISA on beads for quantification of secreted IL17

Cells were sorted to SPR, DP and SPF as described above. For restimulation, sorted cells were plated inside pre-coated wells with 3μg/ml anti-CD3 (Biolegend) and incubated at 37℃ for 4 hr. Plate was centrifuged at 300Xg for 5 min. Supernatants without cells were collected to a fresh plate, stored at -20℃ and thawed at R.T prior to ELISA. Quantitative evaluation of protein levels in supernatant was done using an extension of the ELISA assay, with fluorescent beads (Spherotech PAK) as the solid phase. Primary antibodies anti-IL17A were covalently linked to PE-conjugated beads. Coated beads were incubated with the supernatant and secondary biotinylated antibodies for 2 hr, washed, and stained with streptavidin-PE. A standard curve was generated and fitted using a four-parameter hill function and was used to quantify fluorescent results. Beads were analyzed using LSRII cytometer (BD).

Quantitative RT-PCR

Naive cells were isolated from spleens of mice using magnetic microbeads (Miltenyi Biotec) and underwent Th17 or Treg cell differentiation for 3 days as described above. For the Naive reference sample, naive cells were freshly isolated. Total RNA was isolated using RNeasy mini kit according to the manufacturer's protocol (Qiagen) from 5x10^6^ cells per sample and treated with Turbo DNase to avoid DNA contamination (Ambion). The total RNA was reverse-transcribed into cDNA using M-MLV RT primed with oligo(dT) primers (Promega). Real-time PCR was performed using the Fast SYBR Green master mix (Applied Biosystem) in the LightCycler 480 II (Roche). See supplementary table 2 for primers specification. Primer amplification efficiency and specificity were verified for each set of primers at a final concentration of 250 nM. The determined amounts of cDNA template were 4ng. mRNA expression levels of the tested genes relative to HPRT were calculated using the ΔΔCt method, with the naïve sample as a reference.

**Table S2.** qRT-PCR primers table

| Target | Forward primer | | Reverse primer | | Amplicon size (bp) | Primer source |
| --- | --- | --- | --- | --- | --- | --- |
|  | Sequence 5'-3' | Tm  (℃) | Sequence 5'-3' | Tm (℃) |  |  |
| *Hprt* | AGCCTAAGATGAGCGCAAGT | 58 | TTACTAGGCAGATGGCCACA | 58 | 103 | [(36)](https://paperpile.com/c/liLVgS/ynuF) |
| *Foxp3* | GGCCCTTCTCCAGGACAGA | 62 | GCTGATCATGGCTGGGTTGT | 60 | 112 | [(36)](https://paperpile.com/c/liLVgS/ynuF) |
| *Rorc* | CGCACCAACCTCTTTTCACG | 62.4 | GCTCCATGAAGCCTGAAAGC | 60.8 | 145 | [(5)](https://paperpile.com/c/liLVgS/OXAM) |
| *Il17a* | CTCCAGAAGGCCCTCAGACTAC | 66 | AGCTTTCCCTCCGCATTGACACAG | 67 | 141 | [(37)](https://paperpile.com/c/liLVgS/z9CRi) |
| *Il23R* | GCCAAGAAGACCATTCCCGA | 60 | TCAGTGCTACAATCTTCAGAGGACA | 64 | 81 | [(38)](https://paperpile.com/c/liLVgS/ZL3HM) |

RNA preparation, library construction, and RNA sequencing

Naïve CD4^+^ T cells underwent differentiation towards Th17/Treg cells for 72 hr and were sorted to SPR, DP and SPF cells. Bulk RNA from 150x10^5^ cells per sample was further extracted using the RNeasy micro kit (Qiagen). Libraries were constructed according to the MARS-seq (massively parallel RNA single-cell sequencing) protocol  [(39)](https://paperpile.com/c/liLVgS/QTJLb). Three replicate libraries were prepared for each of the different populations. DNA libraries were sequenced on an Illumina NextSeq 500 with 1-4.5×10^6^ aligned reads per sample.

RNA-seq data analysis

Data was mapped to MM10 mouse database, where 24,063 genes have sequenced reads. Each sample was normalized by its total reads (read/total*10^6^). All low-read genes were filtered out (max normalized read <=10), and ended with 10,005 genes. Read values were converted to Log2. The three biological repetitions were averaged. Correlation matrix between SPR, DP and SPF samples was plotted based on Spearman's rank correlation coefficient. To compare RNA expression levels between DP cells and the two SP populations (SPR and SPF), a similarity index was calculated for each gene. It defines the distance of expression value in DP cells from the averaged expression value of SPR and SPF cells, normalized to half of the range defined by the difference in expression between SPR and SPF cells.

**Equation 1.** Similarity index = DP-[(SPR+SPF)/2](SPR-SPF)/2

All genes with variable expression levels (CV >0.2) were labeled according to their similarity indices: indices in the range of 0.5 to 1.5 were labeled as SPR-like expression; indices in the range of -1.5 to -0.5 were labeled as SPF-like expression; indices in the range of -0.5 to 0.5 were labeled as intermediate expressions. Genes with indices above 1.5 and below -1.5 (“edges”) were uniquely expressed in DP cells. Venn diagram was plotted according to Oliveros, J.C. (2007-2015) Venny, an interactive tool for comparing lists with Venn's diagrams (<https://bioinfogp.cnb.csic.es/tools/venny/index.html>).

Cell loading and culture in micro-well Arrays

We developed a micro-wells system in our lab. We design the array mold, fabricate it using soft lithography, and place it at the bottom of an optical 96-well plate. The array is made from a thin layer of PDMS, which is cheap, easy to mold, non-toxic to cells, and has excellent optical properties. This platform enables the capture and growth of many primary T cells with minimal perturbation, allowing for long-term monitoring of cell activation and differentiation using time-lapse microscopy [(28,29)](https://paperpile.com/c/liLVgS/zC3an+fVvyU). Cells are grown separately inside the micro-wells, but the growth medium is shared, enabling cytokine-mediated intercellular communication. Naïve CD4^+^ T cells were purified from mice spleens using magnetic microbead negative separation (Naïve CD4^+^ isolation, StemCell Technologies). Micro-wells of 25µm diameter were used. To facilitate cell loading into the small-volume micro-wells and eliminate trapped air bubbles that remained in the micro-wells due to the hydrophobicity of PDMS, wells were filled with 200𝜇l of culture medium, and the plate was placed in vacuum for 1 hour followed by 1 min centrifugation at 300Xg to remove residual bubbles. The medium was removed and replaced with 25x10^5^ primary naïve T cells in 100𝜇l of culture medium identical to that used for bulk culture, but without phenol red. The plate was centrifuged at 300Xg for 1 min to allow cells to settle. Residual cells were aspirated and activating anti-CD3 anti-CD28 beads were loaded into the micro-wells in 200𝜇l of medium at a 1:1 bead:cell ratio. Beads were left to settle for 7 min at 37°c after which the medium was aspirated and replaced with 250𝜇l of fresh culture medium supplemented with Th17 or Treg-driving cytokines.

Live cell imaging acquisition

For a time-lapse experiment, a Ti-eclipse microscope (Nikon) was used, equipped with an automated stage, incubator, and a closed chamber that allowed for CO_2_ flow over the 96-well plate. Cells were imaged using 20×/NA = 0.17 objective (sFlour, Nickon) and monitored using bright-field illumination and two fluorescence channels: FITC and Texas Red (for the detection of GFP and RFP, respectively). Time-lapse movies were collected using the NIS elements software (Nikon). Cells were imaged every 30 min for a total of 52 hr, using an Andor iXon-897 EMCCD camera (512 × 512 pixels).

Image pre-processing

Using ImageJ software (https://imagej.nih.gov/ij), rigid registration was applied on images to minimize stage movements effect on deviations in xy position between images. Downstream analysis was further made in Matlab (Mathworks). Images were preprocessed to subtract the background and correct for non-uniform illumination across the field of view. The sum of fluorescence within each micro-well was quantified for each time frame, composing the clonal trace throughout the experiment. The average of five first time frames was subtracted from each trace. Manual tracking was done to filter data for micro-wells that at the beginning of the experiment contained exactly one naive cell with negative fluorescence and 1-3 activation beads. The first division for all micro-wells was detected manually.

Image analysis

Response thresholds for RORγt^GFP^ and Foxp3^RFP^ were defined as elevation above the corresponding fluorescence level of naive cells at the beginning of the experiment. Rise time is the time point when a fluorescent trace crosses the response threshold. For the quantification of response throughout the experiment (fig. S3B), traces that crossed the response threshold for every five sequential time frames (2hr) were defined as positive for the relevant reporter. Definitions for micro-wells’ clonal state were based on traces on the last time frame (t=52hr): SPR clones elevated only GFP signal above its threshold, SPF clones elevated only RFP signal, and DP clones elevated both GFP and RFP signals above their thresholds.

Pixel angels analysis: For each micro-well in a given time frame, we plotted RORγt (y-axis) vs. Foxp3 (x-axis) pixels after masking out beads from the image. Then, for each pixel we calculated its angle between RORγt (90°) to Foxp3 (0°). Pixel angels distribution was smoothed using a gaussian filter to enable local maxima peaks detection above a certain prominence.

Statistics

For all assays, 3 biological repeats were conducted with 3 technical repeats for each, unless noted else. Two-samples t-test was done to validate statistical significance. Statistics were annotated as follows: n.s Pv>0.05; * Pv≤0.05; ** Pv≤0.01; *** Pv≤0.001.

| 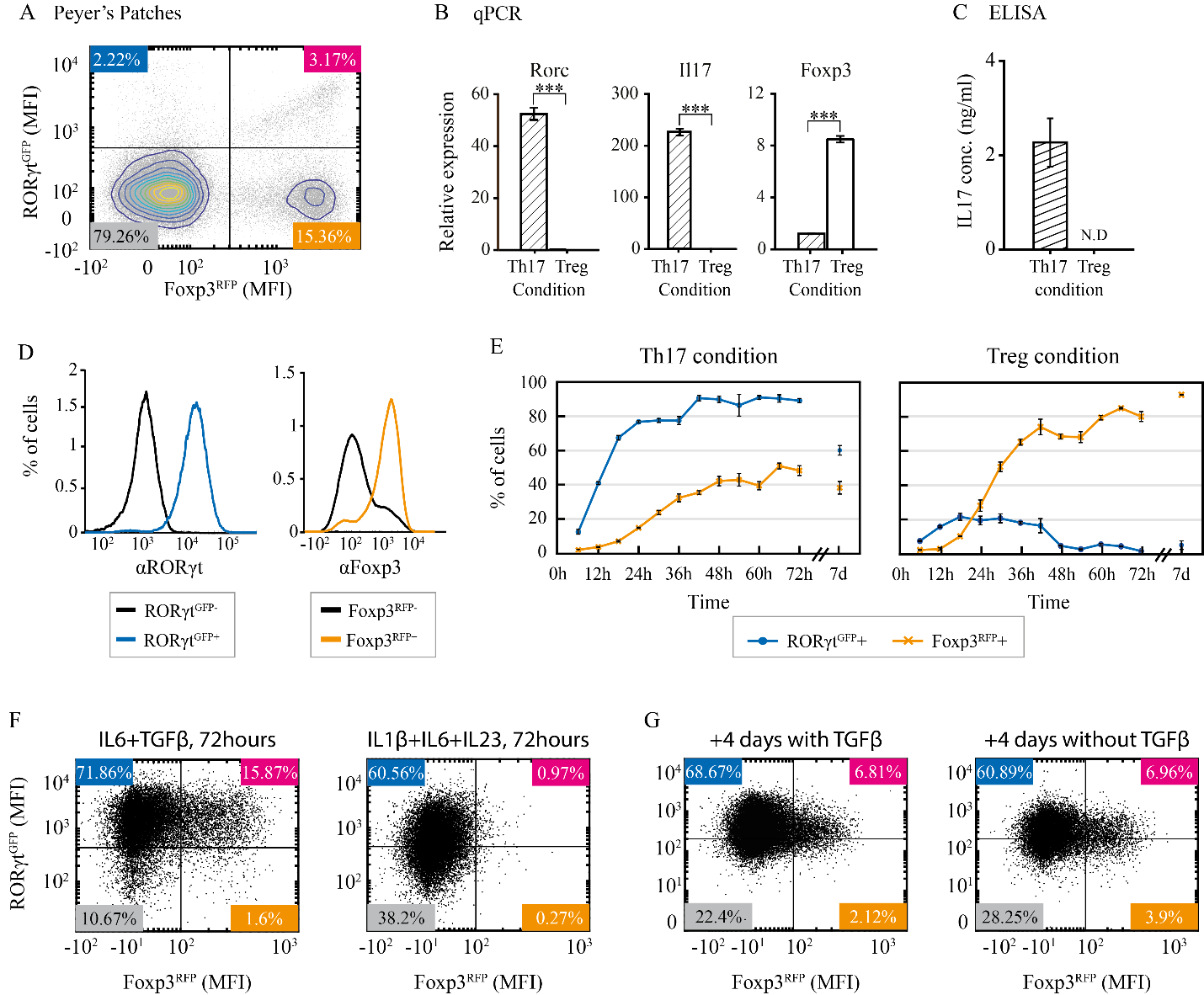 |
| --- |
| **Supplementary Figure 1. DP cells can be found in vivo and emerge dynamically during *in vitro* differentiation. (A)** Scatter plot showing measured expression patterns of RORγt^GFP^ and Foxp3^RFP^ of CD4^+^ T cells that were isolated from Peyer’s Patches of a naïve dual-reporter mouse, and were analyzed by Flow cytometry. One representative experiment out of two. **(B)** mRNA levels of Rorc, Il17a and Foxp3 measured by qRT-PCR on bulk RNA extracted on day 3 of Th17 and Treg cell differentiation. The presented data is from one experiment, 3 technical repeats and is a validation of RNA-seq. Two-samples t-test of technical repeats, Pv≤2.75x10^-5^.  **(C)** Levels of secreted IL17A cytokine were detected using ELISA on beads in cells cultured for 5 days under Th17 or Treg condition. The presented data is from one experiment, 3 technical repeats. **(D)** Histograms of RORγt and Foxp3 levels measured on day 3 of differentiation by flow cytometry. Naïve cells were isolated from RORt^GFP^ X Foxp3^RFP^ F1 generation (left plot) and Treg cells from Foxp3^RFP^ mouse (right plot), differentiated towards Th17, sorted according to their reporters’ signals, and then stained with αRORγt and αFoxp3 antibodies. Black, negative to reporters’ signals; blue, RORγt^GFP+^; orange, Foxp3^RFP+^. One technical repeat out of two is presented. **(E)** Same data as in Fig. 1E. Percentage of cells expressing RORγt^GFP+^ (in SPR or DP) or Foxp3^RFP+^ (in DP or SPF). Measurements were taken every 6hr under Th17 (up) and Treg (bottom) conditions for 72hr and after 7 days using flow cytometry. Blue, RORγt^GFP+^; orange, Foxp3^RFP+^. Presented data is from one representative experiment out of three, three technical repeats in each. **(F-G)** Scatter plots showing measured expression patterns of RORγt^GFP^ and Foxp3^RFP^ of naïve cells that were isolated from splenocytes of 3 mice, pooled and cultured under Th17-inducing conditions. (F) Cells after culturing for 3 days under the standard Th17-inducing protocol with TGFβ (left) or an alternative protocol without TGFβ (right). (G) Cells were cultured for 3 days under the standard Th17-inducing conditions with TGFβ, washed and re-cultured for another 4 days with (left) or without TGFβ (right). Presented data is from one representative technical repeat out of three. |

| 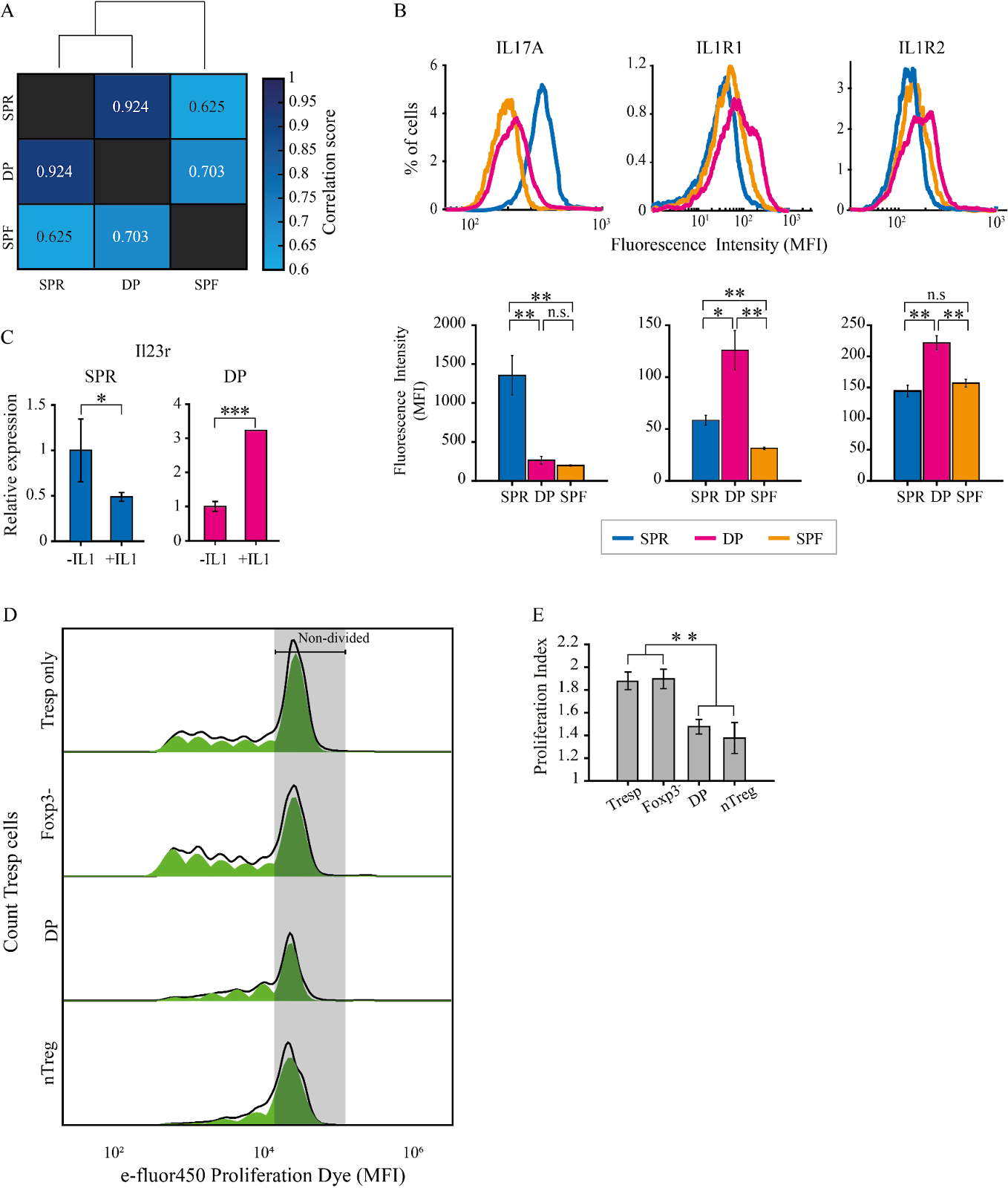 |
| --- |
| **Supplementary Figure 2. DP cells show suppressive characteristics. (A)** Correlation matrix and hierarchical cluster dendrogram based on overall RNA expression profiles showing similarity between the sub-populations SPR, DP and SPF cells after 3 days of differentiation. Shades of blue represent Spearman's rank correlation coefficient Pv. RNA-seq data is from 3 independent biological repeats. **(B)** Histograms of intracellular IL17A levels on day 5 of differentiation (left) and surface IL1R1 and IL1R2 levels on day 3 (right) were measured by flow cytometry (upper panel). Average and standard deviation of the fluorescence intensity for each of the surface proteins is shown (lower panel). Blue, SPR; pink, DP; orange, SPF. Two-samples t-test, IL17 SPR vs. either DP or SPF, Pv=0.01; IL1R1 SPR vs. DP Pv=0.02, SPF vs. either SPR or DP, Pv≤0.007; IL1R2 DP vs. either SPR or SPF Pv=0.01. The presented data is of one experiment with two or three technical repeats. **(C)** Cells were differentiated for 3 days under Th17 condition, sorted to SPR (left) and DP (right) populations, and cultured with or without the addition of IL1 to the growth medium. RNA levels of IL23R measured by qRT-PCR on bulk RNA extracted on day 5 are shown. Two-samples t-test, SPR Pv=0.0161; DP Pv=1.1183x10^-4^. The presented data is an average of three technical repeats from one experiment. **(D)** Histogram of proliferation dye levels measured on day 4 of suppression assay, showing count of Tresp cells cultured either alone (top plot), or with Foxp3^-^, DP or nTregs cells at a ratio of 1:1. Green peaks are generations detected by the FlowJo proliferation model. **(E)** Proliferation index for Tresp cells was calculated as the total number of divisions divided by the number of cells that went into division. Two-samples t-test, Pv≤0.0023. The presented data is of two or three technical repeats from one experiment. |

| 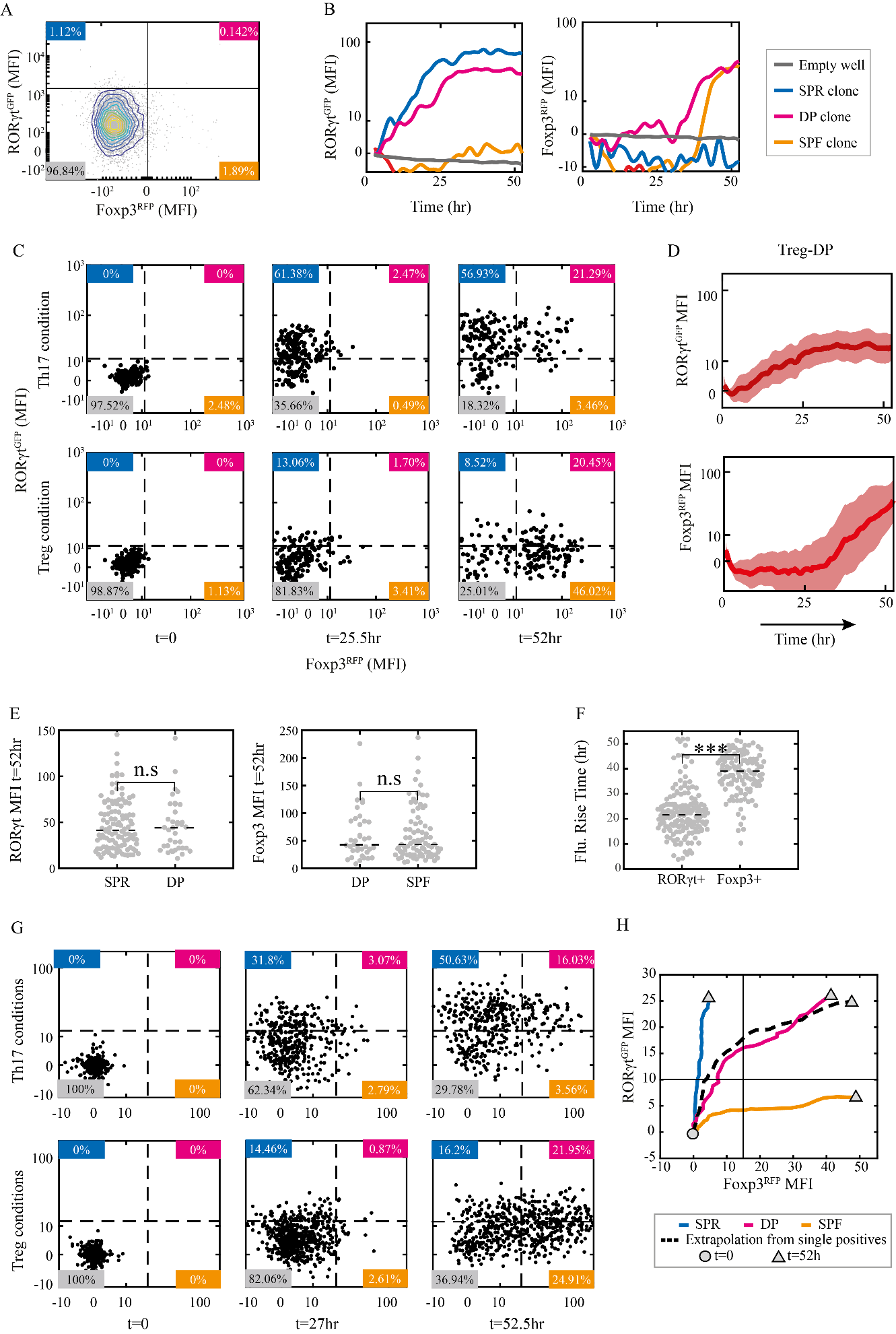 |
| --- |
| **Supplementary Figure 3. Detailed dynamical properties can be extracted from micro-well based time-lapse data.** Naive cells were cultured inside micro-wells, supplemented with differentiation-driving cytokines and monitored under fluorescent microscopy for 52hr. **(A)** Scatter plot showing measured expression patterns of naive cells isolated from RORγt^GFP^ X Foxp3^RFP^ Fl generation using Stem Cell kit. **(B)** Fluorescent traces of RORγt^GFP+^ (left) and Foxp3^RFP+^ (right) over time in an empty micro-well (gray line) or exemplary clones for SPR, DP and SPF (blue, pink and orange lines, respectively). **(C)** Scatter plots showing measured expression patterns of RORγt^GFP^ and Foxp3^RFP^ of wells under Th17 (upper panel) and Treg (lower panel) conditions on t=0,25.5,52hr. Population color code: blue, SPR ; pink, DP ; orange, SPF ; gray, DN (double negative). Dashed line, response threshold determined by clones at t=0. **(D)** Median values of traces over time of RORγt^GFP^ (top) and Foxp3^RFP^ (bottom) in DP clones under Treg condition. A median of data is shown ±std. **(E)** Fluorescence intensity of RORγt^GFP^ (left) and Foxp3^RFP^ (right) in SP vs. DP clones at the last time frame (t=52hr) of micro-wells experiment. Each dot represents one micro-well. Dashed line, median values. Two-samples t-test, Pv>0.05. **(F)** Fluorescence rise time of RORγt^GFP+^ clones under Th17 condition and Foxp3^RFP+^ clones under Treg condition. Each dot represents one micro-well. Dashed line, median values. Two-samples t-test, Pv=7x10-32. The presented data is from one representative experiment out of two. **(G-H)** Repeats for supplementary fig. 3C & Fig. 3D (respectively) using data analyzed from an additional MWA experiment. In this experiment, 1092 naive cells were monitored for 52hr upon differentiation towards either Th17 or Treg cells. (G) Scatter plots showing measured expression patterns of RORγt^GFP^ and Foxp3^RFP^ of wells under Th17 (upper panel) and Treg (lower panel) conditions on t=0,25.5,52hr. (H) Median values of traces of SPR, DP and SPF clones drawn in the space of RORγt (y-axis) vs. Foxp3 (x-axis). Blue, SPR; pink, DP; orange, SPF. Dashed line, extrapolation of median values of RORγt in SPR clones vs. median values of Foxp3 in SPF clones over time. Circular node, t=0; triangle, t=52hr. |

| 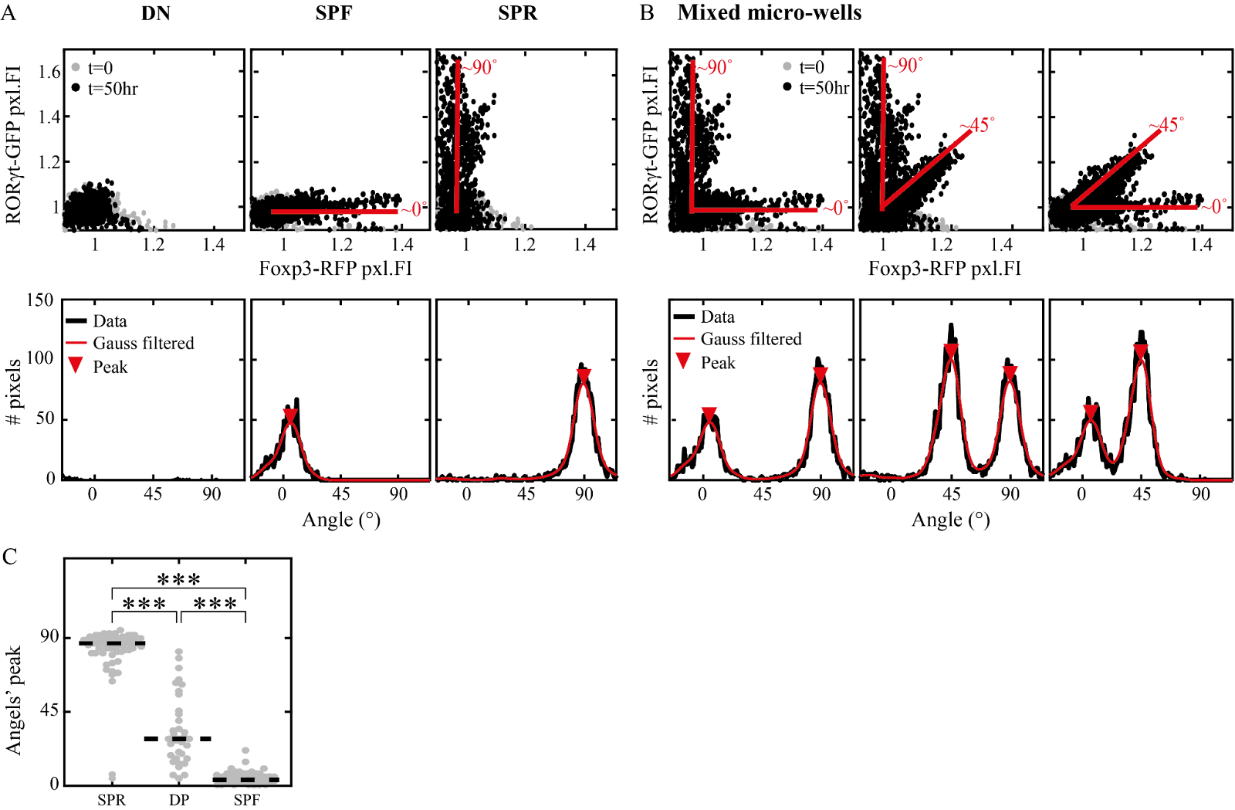 |
| --- |
| **Supplementary Figure 4. Clones during differentiation exhibit homogeneous behavior.** **(A-B)** Pixel fluorescent intensity angles between RORγt^GFP^ (90°) and Foxp3^RFP^ (0°) on t=0 and t=52hr (gray and black dots, respectively; upper panel) and distribution of pixel angles over the last 5 time frames (lower panel) in representative examples of DN, SPF and SPR clones (A) and an artificially mixed micro-wells (B). Upper panel - red line, vector of the detected angles. Lower panel - red line, gauss filter;  red triangle, automatically detected maxima local peak. Each dot represents one pixel. **(C)** Angles’ peaks found over distribution of the last five frames in SPR, DP and SPF clones. Dashed line, median values. Each dot represents one micro-well. Two-samples t-test, Pv≤6.6x10-23. Presented data is from one representative experiment out of two. |

| **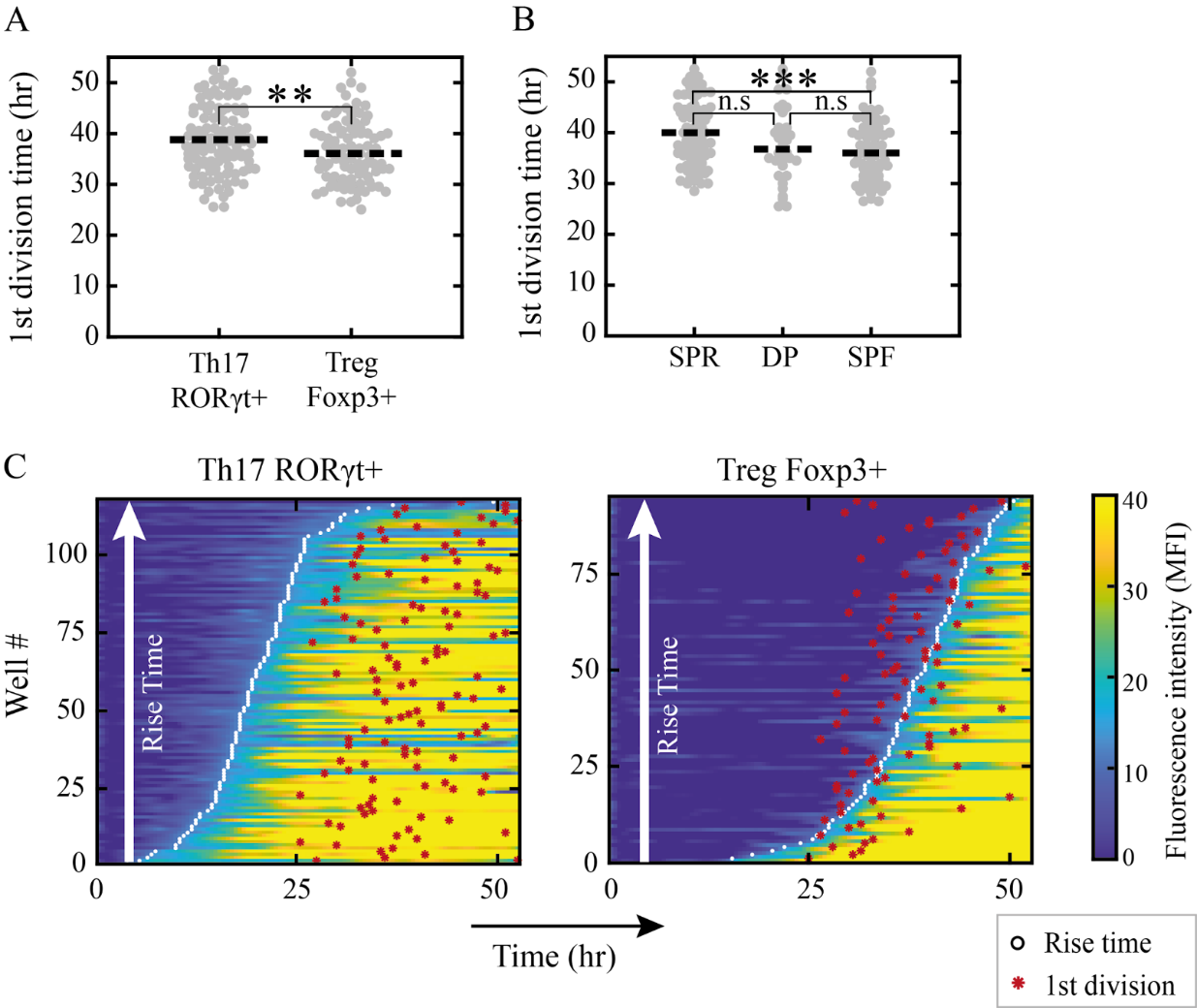** |
| --- |
| **Supplementary Figure 5. Multicellular clones change their phenotype in a synchronized manner. (A-B)** First division time in dividing RORγt^GFP+^ clones under Th17 condition and Foxp3^RFP+^ clones under Treg condition (A) and in dividing SPR, DP and SPF clones (B). Each dot represents one micro-well. Dashed line, median values. Two-samples t-test, Th17 vs. Treg Pv=0.005 (A), SPR vs. DP Pv=0.09, SPR vs. SPF Pv=9.84x10^-4^, DP vs. SPF Pv=0.37 (B). **(C)** Expression over time of RORγt^GFP+^ under Th17 condition (left) and Foxp3^RFP+^ under Treg condition (right) in dividing clones. Each row represents 1 micro-well, color is indicative of expression level (fluorescence intensity). White dot, rise time; red asterisk, first division time. Presented data is from one representative experiment out of two. |

| 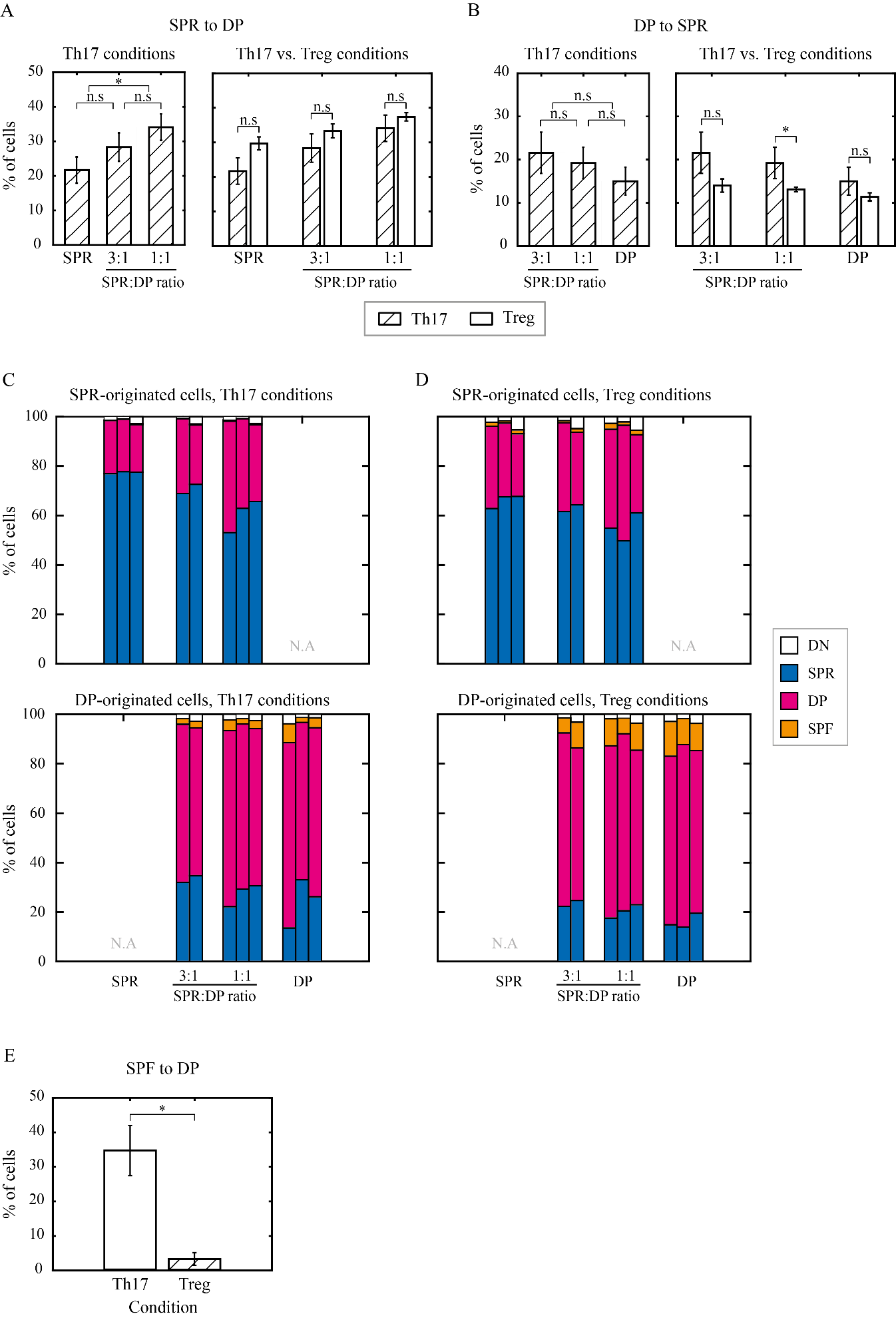 |
| --- |
| **Supplementary Figure 6. Environment shows minimal effect on differentiation state after long incubation. (A-B)** Statistical test for the data presented in Figures 4C, D. Cells were cultured under Th17 conditions for 2-3 days. Then cells were sorted to SPR and DP sub-populations and re-cultured for additional 2 days, either individually (100% SPR; 100% DP) or co-cultured at 3:1 (75% SPR:25% DP) and 1:1 (50% SPR:50% DP) ratios. Resulting populations are presented: SPR originated cells becoming DP cells (A) or DP originated cells becoming SPR (B). Right graphs, resulted populations cultured under Th17 conditions; left graphs, resulted populations cultured under Th17 vs. Treg conditions. Two-samples t-test is shown, Pv=0.049. The presented data is from three independent biological repeats. **(C-D)** Sorted SPR and DP cells were re-cultured for additional 4 days (day 6 or 7 from the beginning of experiment) individually or co-cultured at 3:1 and 1:1 ratios. Resulting populations: SPR originated (upper panel) or DP originated cells (lower panel) that were re-cultured under Th17 (C) or Treg (D) conditions were measured on flow cytometry. Three or two independent biological repeats are presented. Blue, SPR; pink, DP; orange, SPF; white, DN. **(E)** Statistical test for the data presented in Figure 4E. SPF cells were sorted after 3 days of differentiation and re-cultured for 2 days under Th17 or Treg condition. Resulting population: SPF originated cells becoming DP cells. Two-samples t-test, Pv=0.019. Presented data from three independent biological repeats. Oblique line bar, Th17 condition; transparent bar, Treg condition. |
